# Uncovering the differences in linguistic network dynamics of book and social media texts

**DOI:** 10.1186/s40064-016-2598-2

**Published:** 2016-06-24

**Authors:** İlker Türker, Eftal Şehirli, Emrullah Demiral

**Affiliations:** Department of Computer Engineering, Faculty of Engineering, Karabük University, Karabük, Turkey

**Keywords:** Complex networks, Linguistic networks, Social media, Linguistic evolution

## Abstract

Complex network studies span a large variety of applications including linguistic networks. To investigate the differences in book and social media texts in terms of linguistic typology, we constructed both sequential and sentence collocation networks of book, Facebook and Twitter texts with undirected and weighted edges. The comparisons are performed using the basic parameters like average degree, modularity, average clustering coefficient, average path length, diameter, average link weight etc. We also presented the distribution graphs for node degrees, edge weights and maximum degree differences of the pairing nodes. The degree difference occurrences are furtherly detailed with the grayscale percentile plots with respect to the edge weights. We linked the network analysis with linguistic aspects like word and sentence length distributions. We concluded that linguistic typology demonstrates a formal usage in book that slightly deviates to informal in Twitter. Facebook interpolates between these media by the means of network parameters, while the informality of Twitter is mostly influenced by the character limitations.

## Background

A network is a system consisting of components named as nodes, those are interconnected with links. As an emerging branch of science, complex network studies have covered a wide range of applications since the beginning of this century. The early study of Milgram (1967), with a social science perspective, defined the society as a complex network with nodes as individuals and links as relations between them. This study was the first that uncovered the “small world” phenomenon outlining there is a relatively short distance between two nodes in a self-organized system, as an average of six links. This study is consistent with many complex networks such as the .NET Messenger service having an average separation of 6.6 (Leskovec and Horvitz 2008) or today’s Twitter or Facebook friendship networks, with a small update having average distances 4.67 (Sysomos 2010) and 4.7 (Ugander et al. 2011) respectively.

Including the leading studies about complex networks in natural sciences, complex systems in an extensive range of variety like the neural networks, power grid networks, transportation networks, scientific collaboration networks, social networks, the network of film actors, time series, linguistic networks reveal the “small world” properties mentioned above (Albert and Barabasi 2002; Barabasi and Albert 1999; Boas et al. 2009; Cavusoglu and Turker 2013, 2014; Huo and Wang 2016; Marwan et al. 2009; Masulli and Villa 2016; Newman 2003; Newman et al. 2000; Perc 2010; Watts and Strogatz 1998). These studies also showed that the distribution characteristics of the node degrees (i.e. number of connections they have) show a power-law decay that means the systems have high number of nodes having few connections, and low number of nodes treating like hubs (with too many connections), whereas the whole degree distribution range is consistent with a linear decay in log–log scale. Several real networks have a power-law consistent degree distributions (given in Eq. 1) with exponents 2 < *γ* < 3 (Clauset et al. 2009).

1 $$p\left( x \right) = x^{ - \gamma }$$

One of the most surprising outlines of complex network studies is universality that the network parameters are similar with each other, independent from what kind of system is studied (Barzel and Barabasi 2013). Networks of diverse systems like social networks, neural networks or linguistic networks expose similar attributions showing that they have similar organizing principles inside.

In this paper, we focus on linguistic networks defined by the usage of human languages. The construction procedures of linguistic networks vary in methodology, resulting several lexical networks based on different relationships, commonly considering semantic or arrangement properties of the words. These networks also show small-world and scale-free properties which cannot be captured by regular or random network models (Jinyun 2007). Linguistic networks provide a new approach to linguistic quantification, where the various motivations of work can be broadly classified into two main categories. The first category involves with explaining the emergence of universal characteristics of languages by focusing on the structural properties of languages, where the second category uses network presentation of languages to develop systems for machine translation, information retrieval etc. (Choudhury and Mukherjee 2009). The former category that we will also focus on, also aim to provide information about language evolution (Steels 2000; Zeige 2015) and also linguistic typology (Gao et al. 2014; Liu and Li 2010). These studies examine texts as networks, with microscopic linguistic units like words as vertices and their relations as edges (Kohler 2014).

As a remarkable illustration of universality in complex systems, linguistic networks derived from several distinct languages display good consistency in the main network parameters, with smart fluctuations driven by the characteristic linguistic differences (Abramov and Mehler 2011; Gao et al. 2014; Liu and Li 2010; Sheng and Li 2009). Cross linguistic comparisons classify languages, while they also study the universals of human language in the scope of modern linguistic typology (Croft 2002; Liu and Xu 2011; Song 2001). Drawing a comparison about main network parameters, these studies lack a reliable repository of natural speech used in daily life (Liu and Li 2010).

As a growing source of daily entries about random daily circumstances, social media can bridge the gap of natural speech repository, since individuals enter texts without formal care. Another advantage of these repositories is the facilitation to capture the contemporary trends in daily speech, which formal texts cannot. So we can rely at a significant level on the social media entries for defining the direction that a language deviates to.

In this study, we focus on analyzing texts written in a particular language (Turkish), from three different sources consisting of book and social media content as Twitter and Facebook entries. By the way, we aim to display the possible differences in complex network parameters and draw a projection on how a language deviates in an unrestricted media.

## Methods

A remarkable fraction of the linguistic network studies involve in collocation networks, where the words are linked to each other if they co-occur in a sequence or collocate in a certain sentence (Choudhury and Mukherjee 2009; Liu and Cong 2013). Consistent with the applied procedures in the recent studies, we constructed both sequent co-occurrence and collocation in a sentence networks for three different sources as a printed story book (named as *Deli Balta*), Facebook and Twitter entries, all in Turkish language. In the sequent collocation networks, the words sequentially following each other bounded by a sentence are connected. In the sentence collocation networks, we connected all the *n* words collocating in a certain sentence to each other with *n*(*n* − 1)/2 links. By the way, we constructed 6 different networks from these three media, which are undirected and weighted. We limited the number of nodes at each corpus to the minimum of the three corpuses as 12,675 words for comparing the networks vigorously. Investigating the common network measures for these distinct networks, we aimed to uncover the differences of language use in formal and informal media.

While the typology in book is naturally expected to be clear-cut, the social media texts include some noise introduced by the usage of smileys, URLs, non-alphanumeric characters, over-repeated characters etc. A preprocessing procedure was employed to refine the texts from the social media to achieve a comparable corpus with the book texts. Also, the Twitter entries of limited length are assumed as a separate sentence even they do not include punctuation.

## Results

### Basic network metrics

We start with presenting the basic network parameters in this section. The network metrics of sequent and sentence collocation networks are listed in Tables 1 and 2 respectively, displaying significant differences. At a first glance, the number of edges in Table 1 seem approximately six times the values in Table 2. This is an expected result since the structure of sentence collocation networks yield *n*(*n* − 1)/2 edges for an *n*-*word* sentence. This number is *n* − 1 in sequent collocation networks. Driven by this more interconnected structure of sentence collocation networks, Table 2 displays significantly greater values in average degree, link weight and clustering metrics, and smaller path length and diameters.

**Table 1:** Metrics of sequent co-occurrence networks

	Book	Facebook	Twitter
Num. of nodes	12,675	12,675	12,675
Num. of edges	31,382	22,719	32,636
Avg. degree	4.952	3.585	5.15
Avg. weighted deg.	6.154	5.366	11.532
Diameter	13	18	20
Modularity	0.456	0.593	0.552
Avg. clustering coeff.	0.038	0.053	0.063
Avg. path length	3.862	4.385	4.214
Avg. link weight	1.245	1.497	2.248

**Table 2:** Metrics of sentence collocation networks

	Book	Facebook	Twitter
Num. of nodes	12,675	12,675	12,675
Num. of edges	176,156	164,243	161,928
Avg. degree	27.796	25.916	25.555
Avg. weighted deg.	35.55	39.995	56.418
Diameter	6	6	5
Modularity	0.248	0.436	0.433
Avg. clustering coeff.	0.822	0.831	0.811
Avg. path length	2.628	2.66	2.771
Avg. link weight	1.282	1.547	2.208

Despite the high clustering (above 0.8) in the second group of networks and very low clustering observed in the first group, the modularity measures in the first group are slightly above the second group. This is a remarkable result that the networks with low clustering yield greater modularity ratios.

**Fig. 1:**
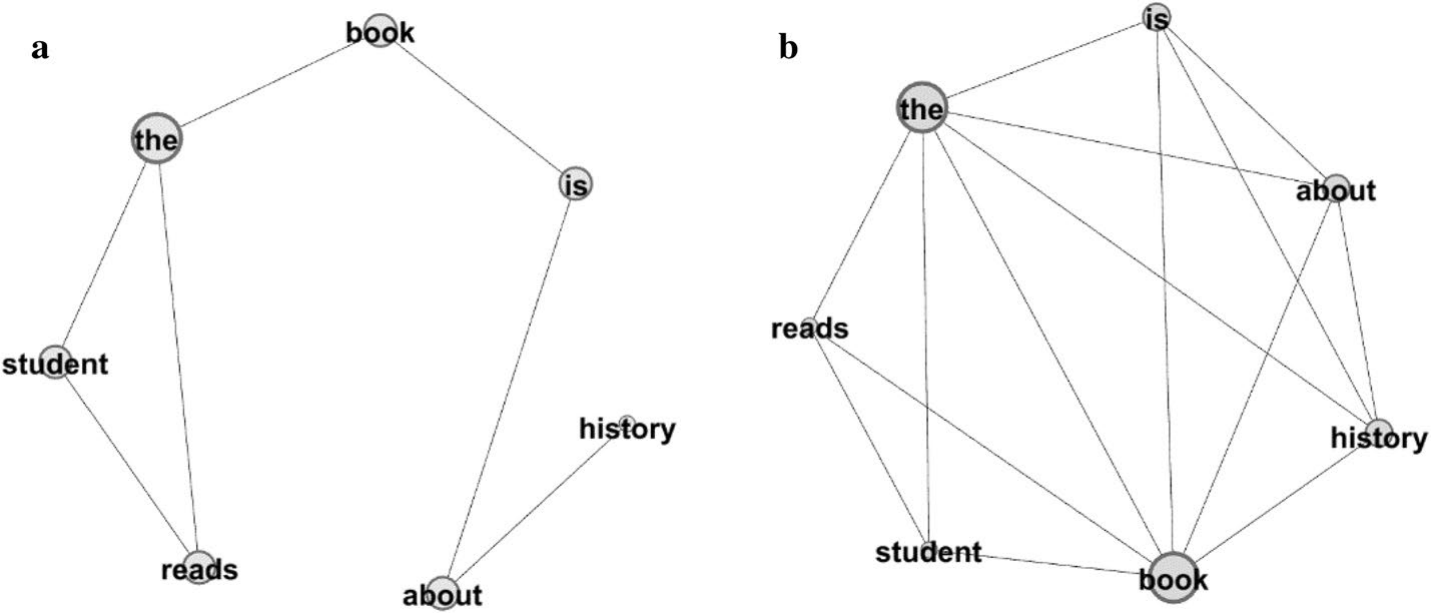
The plots of the **a** sequential, **b** sentence collocation networks. The networks are constructed from the sentences: “The student reads the book. The book is about history”

We can describe this situation by the illustration in Fig. 1. The two network plots in this figure corresponds to the sequential and sentence collocation networks of the same sentences. The size of each node is proportional with the degree (i.e. the number of the neighbors it is connected to) of the corresponding word, while the link weights are ignored since they do not effect clustering and modularity. The network parameters for these networks are given in Table 3. As shown, the sentence collocation network has a higher clustering with very low modularity, since every sentence introduces new and numerous edges between the modules constructed by each sentence. The commonly used words are responsible for this action of incorporating different modules, which in turn marginally reduces modularity measure. The variations of the other parameters listed in Table 1 are also consistent with the main differences between Tables 1 and 2.

**Table 3:** Network parameters of the two layouts in Fig. 1

	Average degree	Diameter	Modularity	Clustering coeff.	Average distance
Sequential	2	5	0.357	0.389	2.429
Sentence	4.3	2	0.080	0.886	1.286

Investigating the three distinct networks of the first group in Table 1, we can say that Facebook network has the minimum average degree and link weight values. The sequent collocation network of the book has the smallest diameter and modularity, yielding more edges between modules that result the smallest path length also. As expected, all the clustering coefficients are very low for these sparse networks. In general, we can conclude that the words in the book texts collocate with the other words more homogenously, resulting shorter distances in the network. The two social media networks also display diversity in the number of edges, average degree and average link weight values, which are notably higher in Twitter network. We suppose that this is a result of the retweeting actions or expressing the feelings in a stereotype manner because of the 140-character limitation, which boost up the weighted degrees and edge weights. As a result, the diameter of the Twitter network remains as the greatest, while having the greatest weighted degree and link weight values.

Investigating the network parameters in the second group given in Table 2, we observe comparable values of edge count, average degree, diameter, clustering coefficient and path length measures. The Twitter network again yields high weighted degree and link weights, reasoned as above. Among the sentence collocation networks, the book network again displays significantly lower modularity, while the clustering is very close to the social media networks. This solidifies our suggestion derived from Table 1 that the word usage book texts are more homogenous, avoiding strict modules by defining numerous interconnections between modules. We can also conclude that, despite having high diameter in sequent collocation networks, Twitter network results a marginal reduction in network diameter in sentence collocation network, possibly originating from the broader usage of some commonly used hub-words in entries that define shorter distances between words.

To conclude the big picture illustrated by Table 2, we can say that from book to Facebook and Twitter networks, less edges with more weights and also more weighted degrees, paired with more modular structures are observed. The remaining parameters preserve the general universal trends, consistent with the small-world property and high clustering. The sequent collocation networks detailed in Table 1 are sparse networks compared to the sentence paired networks as expected. They dominantly have very low clustering and high diameter. To make a distinction among social media texts, we can conclude that Facebook network displays more analogous results with book texts, while Twitter has some exceptional properties mentioned above.

### Degree distributions

To detail the basic parameters that provide a general view on the networks, we present unweighted and weighted degree distributions, and the distributions of the differences of resulting degrees of the connected node pairs in this section.

Degree distributions supply a broad view of the degree occurrence circumstances and provide a classification of the network among the known prototypes. The real networks, including the linguistics generally display generic power-law degree distributions that assign the network as scale-free (Amaral et al. 2000; Newman 2003). A typical power-law distribution equation is given in Eq. 1 below. γ is referred as the power-law exponent which is principally in the interval 2 < *γ* < 3 in most real networks (Albert and Barabasi 2002).

**Fig. 2:**
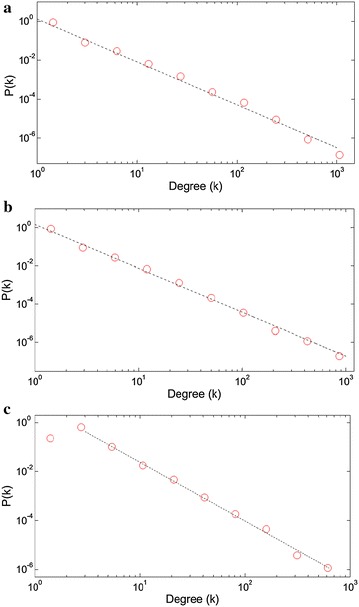
The degree distribution plots with log-binning for **a** book, **b** Facebook, **c** Twitter based sequential collocation networks. The *dotted lines* corresponds to the power law fits in the form of Eq. 1, where γ exponents are 2.2, 2.3 and 2.4 respectively (Clauset et al. 2009)

We start with presenting the degree distribution graphs for the sequent collocation networks of book, Facebook and Twitter texts respectively, in Fig. 2. The degree distributions are all power-law consistent with high accuracy. Only Twitter network yields a short saturation region in the left part. The power-law exponents are 2.2, 2.3 and 2.4 respectively, locating the networks in the ultra-small world part of the scale-free regime (Barabási 2016). The increasing exponents through the social media networks indicate the increasing randomness of the scale-free networks.

**Fig. 3:**
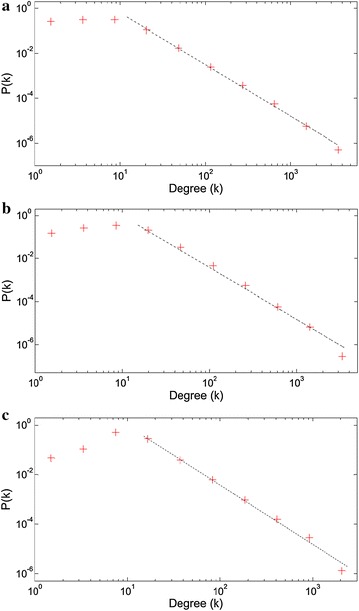
The degree distribution plots with log-binning for **a** book, **b** Facebook, **c** Twitter based sentence collocation networks. The power law exponents γ are 2.3, 2.4 and 2.4 respectively

The degree distributions of sentence collocation networks are presented in Fig. 3. Similar with the sequential collocation pairs, these networks also display power-law degree distributions. But the saturation regimes are evident in the left parts of the plots. The social media based networks exhibit slightly higher power-law exponents (2.4) than the book network (2.3), with more dominant saturation regimes. Consequently, we can say that the book network is organized more systematically among the sentence networks.

Low-degree saturation is a common deviation from the power-law behavior. This indicates that we have fewer small degree nodes than expected for a pure power-law. This saturation is proposed to be caused by the initial attractiveness concept, increasing the linking probability of new nodes to the small-degree nodes, which pushes the small-k nodes towards higher degrees (Barabási 2016). The reflection of this concept to the networks studied here may be explained with the highly connective linking procedure of the sentence collocation networks, which immediately increases the degree of a new node by the number of the words it collocates in a sentence. By the way, the peak *k* value of the distribution graph may give an idea about the mean length of the sentences for that media. This relation will be investigated in the “Word and sentence length distributions” section.

On the other hand, the sequential collocation networks, lacking this highly connective linking structure do not exhibit such a wide saturation region, as presented in Fig. 2.

### Link weight distributions

**Fig. 4:**
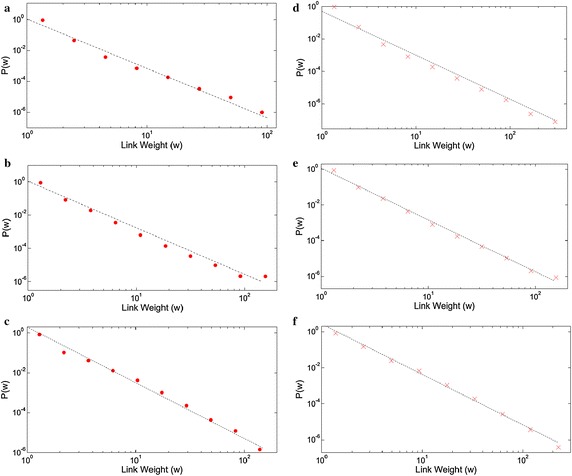
The link weight distribution plots with log-binning for **a** book, **b** Facebook, **c** Twitter based sequential collocation networks. The power law exponents γ are 3.2, 2.8 and 2.8 respectively. The *right side plots* correspond to the link weight distributions for **d** book, **e** Facebook, **f** Twitter for the sentence collocation networks with the exponents γ as 2.7, 2.9 and 2.8 respectively

We also generated the link weight distribution graphs as presented in Fig. 4. These distributions are also power-law consistent, displaying high consistency in the sequential and sentence based versions of Facebook and Twitter texts, all having exponents of 2.8–2.9. While social media networks exhibit similar power-law exponents in both sequential and sentence networks, the exponent of the book text based networks show a steep descent from sequential to sentence collocation networks. This case is dominantly driven by the high exponent observed in the first plot of Fig. 4. Evaluating this case in terms of link weights, we can say that sequent collocation network of book text is more likely to promote low weighted links than the social media sequent networks. Another fact extending this proposal is, the edge weight distribution graphs for sequent collocation networks of social media texts span a weight range up to approximately 200, while this limit is slightly below 100 in sequential book network. On the other hand, this case turns to opposite in sentence collocation networks, yielding weight limits of approximately 200 for social media networks while this limit is 300 for book network. Another possible proposal rises from this fact that book sentence network yields a higher rate of word co-existences in a sentence than the social media texts, promoting the higher link weights than its social media pairs. This may be driven by two facts: Book sentences contain more words or the word collocations in books consist of more predictable and usual combinations that engage the same word pairs more than the social media texts.

### Distributions of the maximum degree differences

**Fig. 5:**
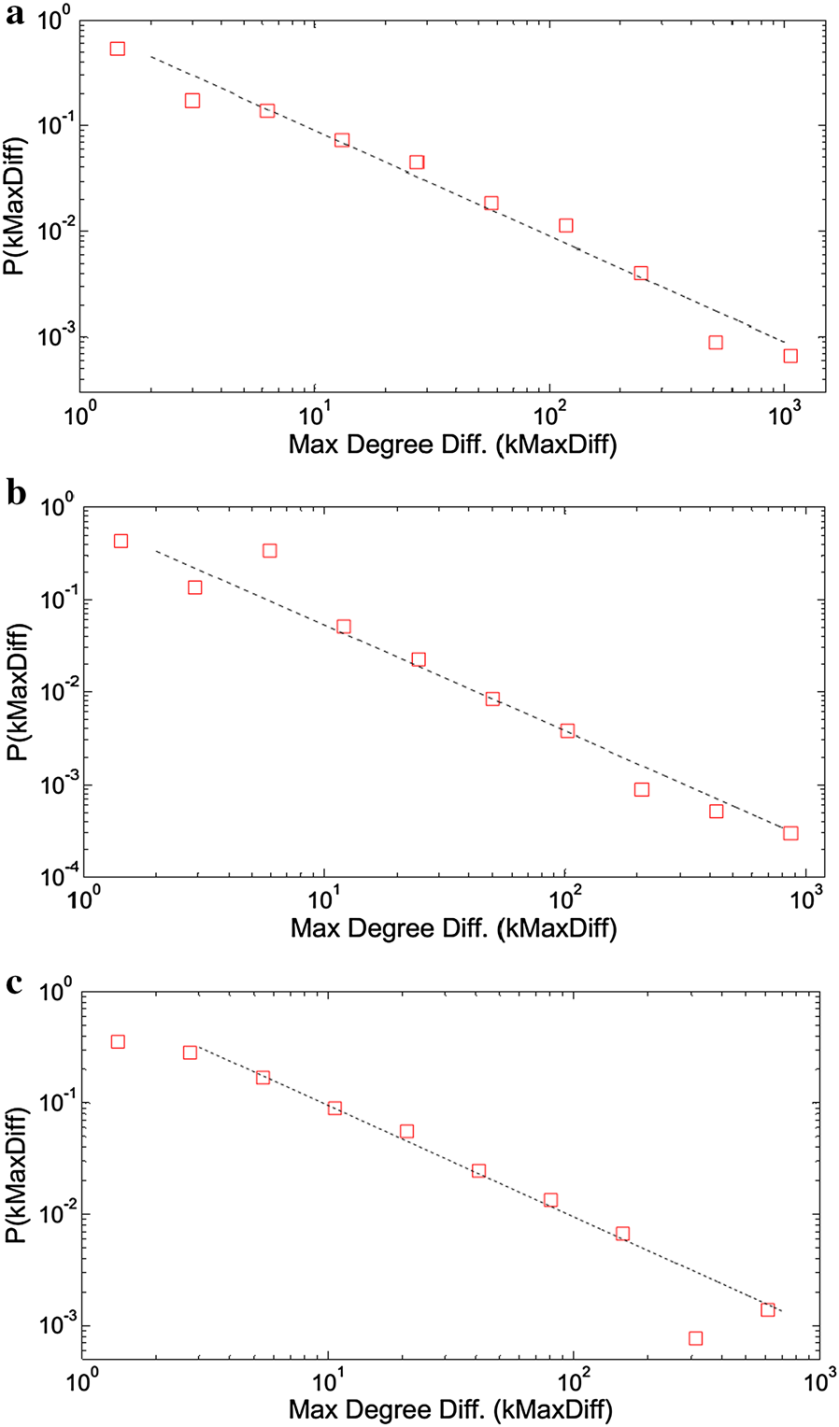
The maximum degree difference distribution plots with log-binning corresponding to the edges of **a** book, **b** Facebook, **c** Twitter based sequential collocation networks. The power law exponents γ are 1.0, 1.15 and 1.0 respectively

**Fig. 6:**
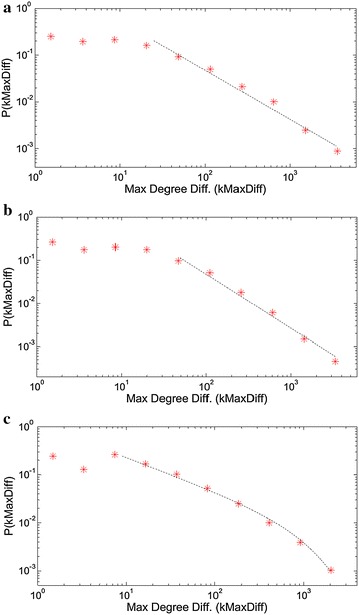
The maximum degree difference distribution plots with log-binning corresponding to the edges of **a** book, **b** Facebook, **c** Twitter based sentence collocation networks. The power law exponents γ are 1.05, 1.25 for the tails in **a** and **b**. The **c** plot is consistent with power-law with exponential cutoff in the form $p\left( x \right) = x^{ - \gamma } e^{{ - \frac{x}{\tau }}}$ having γ = 0.7 and τ = 1150 (Clauset et al. 2009)

To go into the node engagement procedures deeply, we investigated the distributions of the degree differences of the linking pairs. In the edge lists corresponding to each network, we evaluated the final unweighted degrees of each node achieved in the resulting state of the network, and calculated the absolute differences between these maximum degrees. Counting the occurrences of each degree difference value, we converted the data to distribution graphs presented in Fig. 5 (for sequential collocation networks) and Fig. 6 (for sentence collocation networks).

Both sequential and sentence collocation networks exhibit power-law consistent distributions in maximum degree differences, having exponents slightly above 1.0. The sequential collocation networks feature with power-law consistency in the whole distribution, while the sentence collocation networks yield saturation ranges for the low difference values. This difference indicates that the edges in the sentence collocation networks are more likely to engage nodes of comparable degrees. In the other hand, the rarely used words also have comparable degrees with the others because using a word in a sentence rapidly boosts up a node’s degree by approximately one dozen. Among the sentence collocation networks, Twitter network has an exponential cutoff region in the tail, with the cutoff frequency of 1150°. The power-law consistency in various distributions of a system’s ingredients shows that power-laws are everywhere in nature, as the examples mentioned in the previous works (Newman 2005).

### Word and sentence length distributions

Word and sentence lengths are key ingredients of quantitative investigations of human languages (Chen et al. 2015). There are several reviewing articles about word length, which summarize the historical development of word length study, as well as some critical problems concerning the attempt to establish a general law of word length (Chen and Liu 2014). On the other hand, sentence length studies throw light on the understanding of the universalities and peculiarities of human cognitive processes in language as well as language itself (Jiang and Liu 2015).

**Fig. 7:**
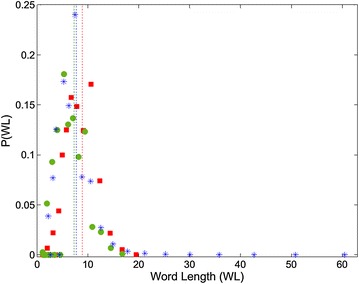
Word length (WL) distribution plots of book (*red squares*), Facebook (*green circles*) and Twitter (*blue* *asterisks*) texts. The mean WL values are also indicated with *vertical lines colored* same with the corresponding *dots*

**Fig. 8:**
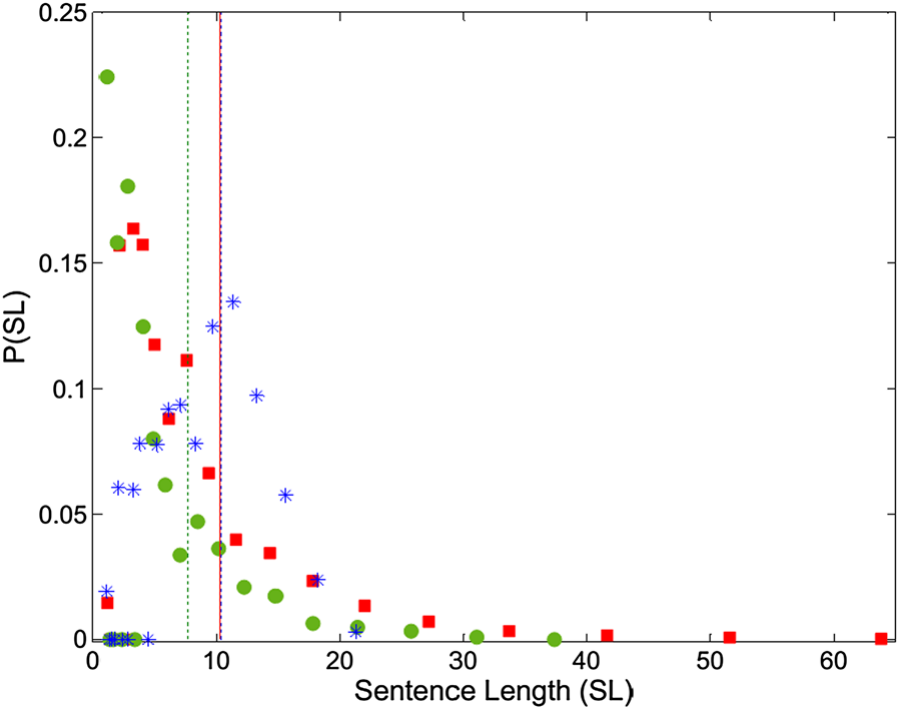
Sentence length (SL) distribution plots of book (*red squares*), Facebook (*green circles*) and Twitter (*blue* *asterisks*) texts. The mean SL values are also indicated with *vertical lines colored* same with the corresponding *dots* (the *vertical lines* for Book and Twitter texts overlap)

To uncover the word and sentence length characteristics of the three corpora we studied, we present the word length (WL) distributions in Fig. 7 and sentence length (SL) distributions in Fig. 8, including the mean length values expressed as vertical lines. The mean length values are also listed in Table 4.

**Table 4:** Average word and sentence length values

	Book	Facebook	Twitter
Average word length	8.899	7.246	7.695
Average sentence length	10.311	7.722	10.393

In Fig. 7, the book and Facebook WL distributions display similar trends, while Twitter is distinctive as it includes WL values exceeding 60 characters. The distributions are also consistent with the recent study of Dalkılıç and Çebi (2003) on WL distributions of Turkish texts, except for the distinct character of Twitter WL distribution. But compared with the WL distributions of other languages (Smith 2012), Turkish words seem to be shorter than the majority (Spanish, Italian, French etc.), and more consistent with Swahili and English languages.

Examining the extremely long words in Twitter, we see that they are not lexically valid words, but are the concatenations of several words (without space characters) to form a *tag*, which is the way of defining and joining the hot topics in Twitter (for ex. the word “29ekimcumhuriyetbayramimizkutluolsun” is used to express the sentence “29 ekim cumhuriyet bayramimiz kutlu olsun” without using spaces). The peak values of the WL distributions, an indicator of the most probable value to occur, is around the mean WL values given in Table 4.

The book texts display the greatest average WL values compared to the social media texts. This may be caused by the law that frequent words tend to be short, that is proposed and popularized by Zipf (1949). The book texts, having a more formal structure, seem to include less popular (and longer) words than the social media texts. In another words, the social media users, expressing their feelings more informally, tend to use more popular (and shorter) words. The slight difference between the Facebook and Twitter WL values is generated by the combined words in Twitter, as mentioned above.

In Fig. 8, the book and Facebook SL distributions again display resemblance, while the book texts include more populated sentences compared to Facebook. This is an expected situation since composing longer sentences require more cognitive processes for the human brain (Jiang and Liu 2015), which is a more suitable behavior for formal texts. The SL distributions are consistent with the recent studies on Turkish language (Örücü 2009), but display peaks for shorter sentences than English and French language (Chen 1993; Jiang and Liu 2015).

The Twitter texts again differ, having a right-skewed SL distribution characteristic. This behavior indicates that Twitter users (despite the informality) tend to compose longer sentences since the character limitation forces them to express themselves in 1 or 2 sentences at most. As a result, Twitter has the greatest SL value in Table 4. In fact, the informal atmosphere of the social media should promote shorter sentences as in Facebook but the peak value in the SL distribution indicates that Twitter users most probably compose sentences of ~ten words, while this peak occurs at lower SL values for book and Facebook texts. The lowest average SL value is achieved for Facebook, which has a similar distribution with book but lacks the high SL region.

In “Degree distributions” section, we proposed that the low-degree saturation region of the degree distributions for sentence collocation networks are correlated with the highly connective linking procedure of the sentence collocation networks. Consequently, the peak *k* value of the distribution graph may give an idea about the mean length of the sentences for that media. Comparing the degree distribution graphs in Fig. 3 with the SL distribution graph in Fig. 8, we observe good consistency in the saturation region (k < 10) and the average SL values. This consistency confirms the reason we proposed for the low-degree saturation regime, which is only evident dominantly for the degree distributions of the sentence collocation networks.

### Maximum degree differences versus link weight percentiles

**Fig. 9:**
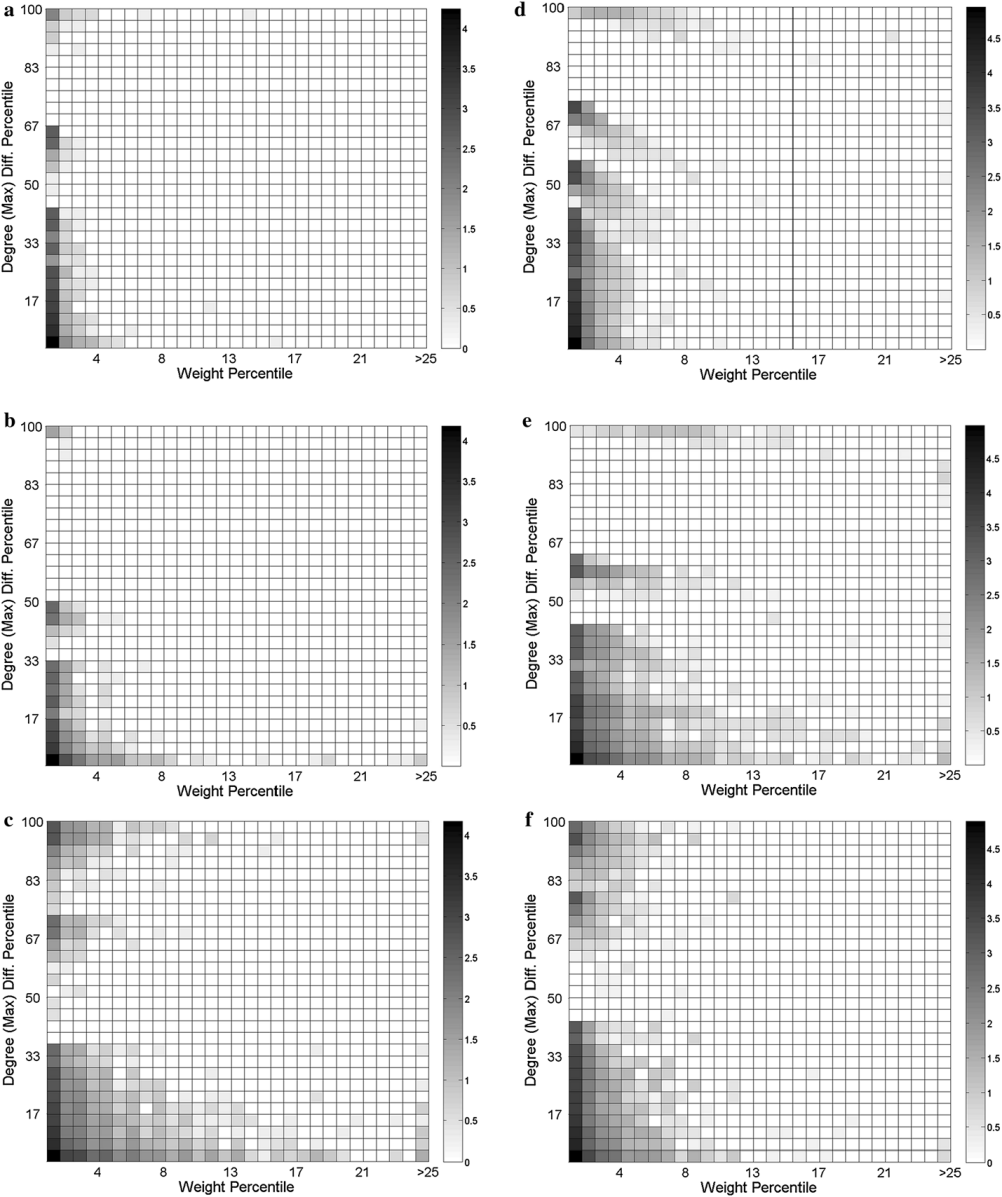
The maximum degree difference versus link weight percentiles of **a** book, **b** Facebook, **c** Twitter based sentence collocation networks. The *right side* percentiles correspond to the sentence networks of **d** book, **e** Facebook, **f** Twitter texts. The two axes are scaled as the percentage of the maximum values of the variables. The *color-bars* are logarithmically scaled for comparative purposes

In the previous sections, we outlined the generic power-law consistency of both link weight and final degree difference distributions. To visualize the engagement tendencies of these two variables, we present 2D coupling percentiles in Fig. 9 with each grid gradually filled as an indicator of the recurrence rate as a third dimension of the graph. The color-bars are also scaled logarithmically, resulting a gray tone equivalent to 2 for the repetition of 100 times for a cell, since log_10_ 100 = 2. The sequential network plots are grouped at left side and sentence network plots at the right side, ordered as book, Facebook and Twitter respectively.

The links of the sequential collocation network of book and Facebook texts (plots A and B) exhibit increasing weight circumstances especially for low degree differences. This means that sequential usage of the words having similar degrees are dominant in these networks. Twitter embraces more usage of popular and unpopular words sequentially, resulting a rate of weighted links in the upper side of plot C.

For the sentence counterpart of the book network (Plot D), the majority of the degree difference range exhibit weighted link occurrences except for a narrow band around 80 %. This indicates that the usage of words having various popularity in a sentence is more frequent in the books, rather than Facebook which has a broader region of empty percentile. On the other hand, Facebook roughly imitates the formation of book percentile with inclined stripes, except for the broader empty region mentioned above and a broader weight percentile in the bottom region. This bottom region seems analogous with the twitter percentile, again positioning the Facebook network as an interpolation between the book and Twitter networks. Twitter sentence network, together with the sequential counterpart (plots F and C), exhibits a diverse scattering character with a notch in moderate range of the degree differences in both plots. This points out that the sentence collocations of words in Twitter consist of either divergent popularity words or similar popularity ones.

The separated uppermost lines of some of the percentile graphs indicate that the uncommon words are most likely to couple with highly popular words, generating the glowing 100 % lines of the degree differences axes.

## Discussion

We have investigated linguistic networks of book and social media texts as Facebook and Twitter, having equal number of nodes (distinct words). The words used in every media are linked by two ways: first, the sequential collocating words and second, the words collocating in a particular sentence are linked to each other. By the way, we achieved six different networks from these three distinct media.

The first group consisting of the networks of sequential collocation has sparse connections compared to the sentence collocation network group as expected, resulting smaller average degree, link weight and clustering metrics and higher path length and diameters. Among these parameters clustering is marginally low, while the second group of sentence collocating networks with more interconnected structures, have remarkably high clustering compared to the first group. Despite this high clustering, the sentence collocation networks typically have smaller modularity measures compared to the sequential collocation networks, possibly caused by the commonly used words that incorporate different modules and reduce modularity. This fact, illustrated in Fig. 1, is also originating from the dense connection procedure of the sentence networks.

Among the sequential networks group, book network has the smallest path length, diameter and modularity, yielding more edges between the modules. The words constituting this network seems to collocate with each other more homogenously. Among the social media networks, Twitter texts have notably higher number of edges, average degrees, link weights, that are possibly originated from both the retweeting actions and expressing feelings in a more expected form due to the character constraint of 140 words.

The sentence collocation networks group yield more comparable values with similar deviations between the three media. The network structures seem to get more informal from book to the Twitter texts, while Facebook interpolates between these media. Less edges with more weights and weighted node degrees are observed from book to Twitter media, paired with more modular structures.

Among the social media texts, Facebook media display more analogous results with the formal media. The universal network characteristics of small world and high clustering are observed rather in the sentence networks. The sparse structure of sequential collocation networks inhibit clustering and small-world properties as expected. However, the degree and link weight distributions, together with the degree difference distributions display coherent power-law distributions for all the six networks. This state indicates that the engagement principles of the language between the words are preserved regardless from the media and usage constrains.

The word and sentence length distributions add depth to the network approach to linguistics. The WL distributions are in good agreement for the three corpora, while the concatenated word combinations in Twitter causes an extensive right region, and an average WL value greater than Facebook. Book texts include longer words in average as a result of the usage of less popular (but longer) words. The SL distributions for book and Facebook texts are again similar, while Twitter distribution is right skewed compared to them. The peak value for Twitter SL distribution is apparently greater, indicating a bias of long sentences caused by character limitation of the media that forces users to express themselves in a sentence. Facebook texts are distinctly shorter in average, indicating that informal sentences are shorter in case of no restrictions applied.

Among the power-law exponents of the link weight distributions, the sequential book network is exceptional with a higher slope of −3.2. Thus, the sequent collocation network of book text is more likely to promote low weighted links than the social media pairs. Another noteworthy result of the edge weight distributions is, the maximum expected edge weights remain comparable (around 150–200) for the sequential and sentence versions social media networks, while this quantity increases approximately from 100 to 300 for the book networks.

The maximum (or resulting) degree difference graphs again display similar characteristics except for the sentence network of Twitter, which yields an exponential cutoff region with a cutoff frequency of ~1150. As a result of this variety, the maximum degree difference scale is limited around 2000 for the Twitter network, while the book and Facebook sequential networks exhibit degree differences up to 3500 s.

Lastly, the maximum degree differences versus edge weight percentile plots display how Facebook networks interpolate between book and Twitter networks again. In the upper half band of the degree differences axes, Facebook imitates the book network with a broader empty region, while the bottom half is very similar with the Twitter plots. Among the percentile plots, Twitter networks exhibit distinct formations with a notch in the central degree difference axes in both sequential and sentence networks, indicating that the co-occurrence of words in Twitter consist of various popularities except for the middle notch of the degree difference axis. To define a characterization between the left and right side percentile plots, we can conclude that the sentence percentiles are the stretched versions of their sequential versions, except for the Twitter percentiles that seem quite analogous with each other. This portrait demonstrates that the node degree and link weight coupling characteristics of Twitter do not deviate in the sequential and sentence network approaches.

## Conclusion

Studied as sequential and sentence collocation networks, the book and social media texts display different characteristics yielding the variations in the language use in formal and informal media. These variations get distinctive by the addition of character limitations in Twitter, and also influenced by the actions of duplicating entries called “retweeting”. So the Twitter part of the study cannot be evaluated as a pointer of the language evolution. But the Facebook texts, written more comfortable without limitations, can be evaluated as the direction of the language evolution. By this point of view, the statistical differences in book and Facebook texts mentioned above define the alternation of the language use in formal and informal media, also defining the deviation that is firstly evident in the informal media.

The statistical differences in linguistic dynamics studied here evaluates the words “as is”, so no further processing like stemming and lemmatization are applied beyond the pre-processing mentioned in the methods section. A further study is planned with enhanced language processing tools to uncover the linguistic deviations in these media more precisely. But we consider that the “as is” approach employed in this study is also valuable since is preserves the word usage behaviors. We also consider that a further challenge about sequential linguistic networks is that they can be employed in semantics, since they preserve the word neighborhood instances. Both directed and undirected network approach should be investigated as an alternative to the bag-of-words semantic applications which do not consider the sequential configurations of words. This is the subject of another further study about linguistic networks, that may also be empowered by stemming and lemmatization.
